# Country size bias in global health: cross-country comparison of malaria policy and foreign aid

**DOI:** 10.1186/s41256-020-00176-x

**Published:** 2021-02-03

**Authors:** Tomas Jezek

**Affiliations:** Masaryk University, Brno, Czechia

**Keywords:** Endemic diseases, Environment and public health, Global burden of disease, Global health, Health care economics and organizations, International agencies, Malaria

## Abstract

**Background:**

Foreign aid has been shown to be favourably biased towards small countries. This study investigated whether country size bias also occurs in national malaria policy and development assistance from international agencies.

**Methods:**

Data from publicly available sources were collected with countries as observational units. The exploratory data analysis was based on the conceptual framework with socio-economic, environmental and institutional parameters. The strength of relationships was estimated by the Pearson and polychoric correlation coefficients. The correlation matrix was explored by factor analysis.

**Results:**

Malaria burden is strongly correlated with GDP per capita, total health expenditure per capita, HDI; moderately with latitude, weakly with elevation, urban population share, per capita funding from the Global Fund, PMI USAID, UK government and UNICEF. Small country status is strongly correlated with population size, land area, island status; moderately with development assistance received per capita, weakly with funding per capita from Global Fund, government NMP and PMI USAID. Policy score 1, a variable derived from our factor analysis and related to malaria endemicity, is significantly strongly correlated with the malaria burden, moderately with HDI, GDP per capita, total health expenditure per capita, PMI USAID funding; weakly with island status, urban population share, latitude, coastal population share, total government expenditure and trade openness, Global Fund funding, World Bank funding, UK government funding, and UNICEF funding per capita. Policy score 2, which captures variation not related to malaria endemicity, is significantly weakly related to the ICRG index, PMI USAID funding per capita and small country status.

**Conclusions:**

The results suggest that malaria burden and economic development are bidirectionally related. Economic development can contribute to a reduction in the malaria burden. Country size does not negatively impact malaria burden, but it does account for greater development assistance per capita from selected international agencies. National malaria policy is associated with parameters related to public governance and is modified in small countries. Small country bias is present in the distribution of socio-economic resources and the allocation of foreign aid. Small countries are characterized by distinct environmental and socio-political properties.

**Supplementary information:**

**Supplementary information** accompanies this paper at 10.1186/s41256-020-00176-x.

## Background

Occurrences of foreign aid can be traced as far back as the nineteenth century. Later analyses of foreign aid flows have revealed that small countries receive more foreign aid per capita than large countries. This fact has been referred to as the “small country effect”. One explanation suggests that the economies of small countries are more open and thus require more aid to finance their imports [1]. Further attempts to explain this bias have used models with socio-economic parameters and have reported, that small country bias is a result of various economic and political properties. Such results have been reflected in the decision-making of international agencies. Voting rights are assigned to a single country regardless of its population size. Therefore it is likely that the political impact of small countries is higher than can be accounted for by their population size.

Studies of the role of geography on economic development have found several associations with gross domestic product (GDP) per capita [2]. Coastal economies are favourably located for foreign trade and generally have higher GDP than landlocked economies. Coastlines and areas connected to the coast by navigable waterways are more densely populated than the hinterlands. Most tropical countries are economically poor.

Global development after World War II has included strategically located colonies gaining independence. Analyses of former colonies have shown that economic development is substantially impacted by institutional development [3]. Some authors suggest, that former colonial rule was replaced by neocolonial practices, which allow former colonizers to exercise indirect economic control by means of financial aid from international organizations, bilateral donors and (semi-)private investors [4]. Other authors consider colonialism to be a direct socio-political determinant of health [5]. Some authors have suggested that a history of socialist systems or conflicts and wars can impact economic development. Further studies suggest the quality of institutions as a fundamental determinant of economic development [3].

The relationship between socio-economic parameters and health constitute one of the foundations of epidemiology as a scientific discipline. Malaria is a tropical infectious disease that still accounts for one of the most deaths globally and has the greatest impact on global public health. Malaria prevention and treatment remains one of the most cost-effective public health interventions. The return on investments in malaria prevention and treatment could be as high as 4000% [6]. A country’s malaria burden is strongly related to its economic development [2]. Poverty increases malaria transmission and malaria causes poverty by impeding economic growth. Policies for malaria control should be included in government agendas for poverty reduction [7]. The relationship among the environment, institutions, disease endemicity and health policy have rarely been explored in cross-country comparisons. Malaria national health policy is defined at the country level so comparisons between countries could provide more insight into the complex process of creating health policy. Foreign aid for malaria control is largely provided by international organizations such as the Global Fund to Fight AIDS, Tuberculosis and Malaria (Global Fund), the World Bank or the United Nations Children’s Fund (UNICEF). Global aid is distributed to countries, thus, governments of small countries may exert a stronger influence on aid allocation than their size would suggest. Better insights into these processes are critical for improving the allocation of foreign aid.

Policies are usually determined through complex interactions among key stakeholders. Little is known about the decision-making process for selecting and altering national malaria intervention policies. The influence of major factors is difficult to identify, but it clearly varies between countries. Robust analytical frameworks are not yet available [8]. Health policy analyses tend to omit the political characteristics of the public health agenda.

Environmental, socio-economic and institutional characteristics are hypothesoized to affect a country’s malaria burden, either directly through their impact on biological vectors or indirectly. The purpose of this study was to identify and understand the key parameters that affect decision making and policy implementation. The results should assist decision makers in critically evaluating public health interventions for malaria control.

## Methods

### Study design

National malaria intervention policy and strategy adoption (Table 5) is assumed to vary across countries. This variability is related to characteristics, that is not known or is not directly observable. Factor analysis allows to identify underlying relationships as it creates new factors with detectable relationships to the latent parameters. Based on these factors countries can be distuinguished based on the suite of health policies they have adopted.

### Model

**Fig. 1:**
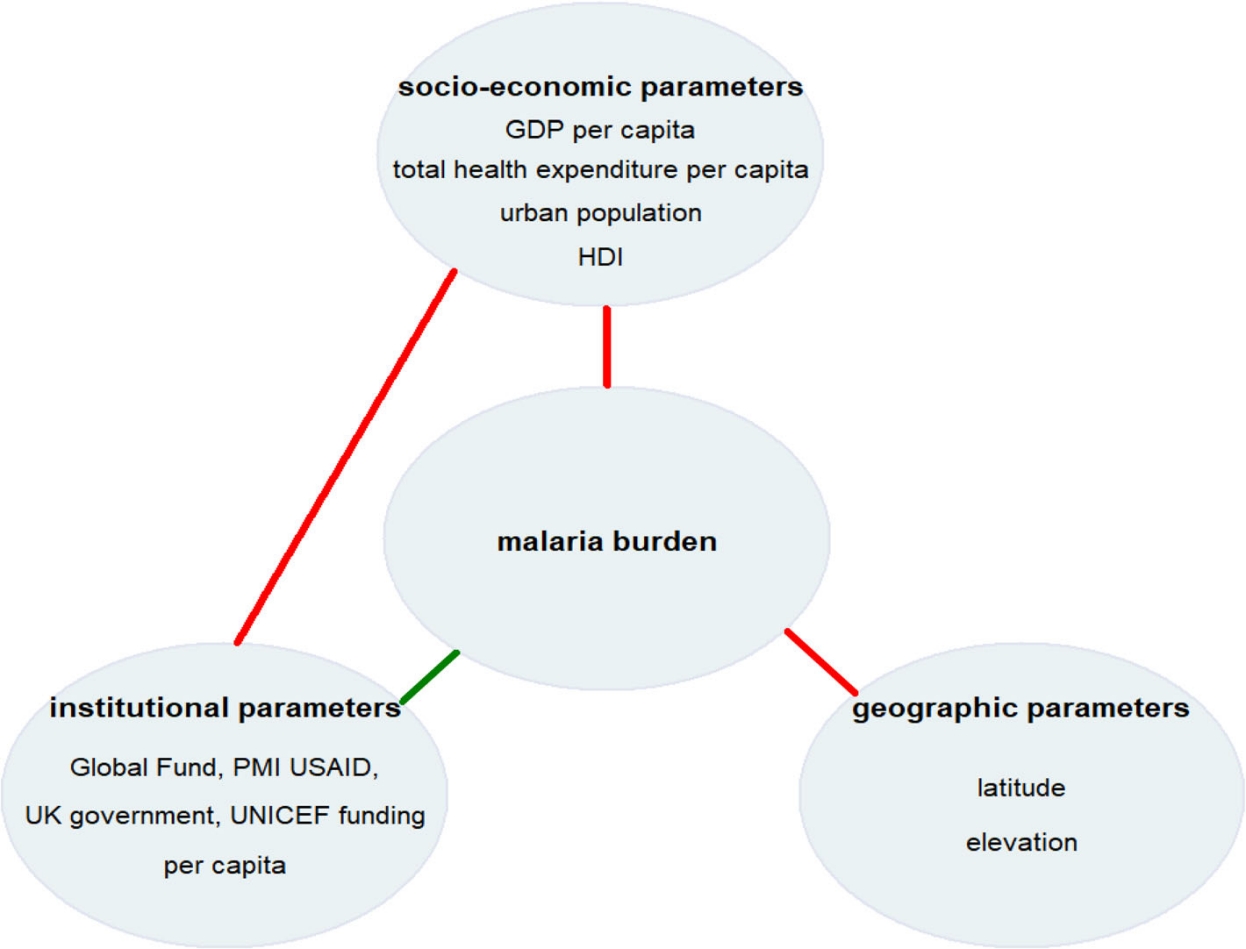
Conceptual framework of malaria burden. * framework categories (socio-economic, institutional and environmental) with corresponding parameters. a green line indicates a positive correlation, a red line indicates a negative correlation of parameters with malaria burden. GDP: gross domestic product; HDI: human development index; PMI: United States President’s Malaria Initiative; UK: United Kingdom of Great Britain and Northern Ireland; UNICEF: United Nations Children’s Fund; USAID: United States Agency for International Development

To address the study questions, a conceptual framework was constructed (see Fig. 1). Based on the literature review parameters at the country level were identified that could be related to the malaria burden. The country unit was used as defined by the International Organization for Standardization [9]. These parameters can be grouped into three categories: (i) socio-economic, (ii) environmental and (iii) institutional. Socio-economic parameters include GDP, the national budget, economic stability, and the level of urbanization. Environmental parameters are related to geographic conditions such as temperature, latitude, elevation, and coastal and island locations. Institutional parameters include governmental policies, trade barriers, health services and policies, educational programmes, agricultural incentives and infrastructure projects [10].

Potential outliers were identified. Quantitative variables with skewed distributions were transformed by the common (base ten) logarithm or by the square root, whichever achieved a better distribution. Variables with negative and zero values were adjusted prior to transformations to avoid data loss.

The strength and the direction of linear relationships between continuous variables was estimated by the Pearson product-moment correlation coefficient (*r*). Magnitudes were interpreted as strong, moderate and weak based on range thresholds of 0.70, 0.50 and 0.20 in absolute values.

The strength and the direction of relationships between categorical variables was estimated by the polychoric correlation coefficient. The tetrachoric variant of the polychoric correlation is used to estimate the correlation between two dichotomous variables. The tetrachoric correlation treats the 2 × 2 contingency table as a double dichotomization of a bivariate standard normal distribution. Karl Pearson considered tetrachoric correlation to be one of his most important contributions to the theory of statistics. However, the method was difficult to compute and was not used [11]. Correlation matrix was created with tetrachoric correlation coefficients, from which factors were extracted. Those factors with the eigenvalue greater than 1 were retained for further analysis (the Kaiser-Guttman rule) [12].

### Variables

The definition of a small country is not standardized. The World Bank uses a population threshold of 1.5 million or a membership in the Small States Forum [13]. Surface area is not used as a stand-alone criterion, whereas other definitions are based on aggregated indicators [14], factor analysis [15] or cluster analysis [16]. A conservative approach used for this study included only countries that fulfil all of the above-criteria except for membership in the Small States Forum. The resulting countries are listed in the Additional file 1.

Openness was measured by the proportion of years within 1965 to 1990 that a country was open to trade [17]. It is a measure of a country’s macroeconomic policies that interfere with a foreign trade.

The international country risk guide (ICRG) rating is a widely used indicator of institutional quality in cross-country comparisons. The index was constructed from five indicators that are supposed to be highly relevant to the security of private property and the enforceability of contracts: the frequency of contract repudiation, the risk of expropriation, corruption in the government, a tradition of law and order, and bureaucratic quality [18]. Countries were scored from zero to ten according to perceived institutional quality (the higher the score, the lower the risk). Data for the ICRG index were taken from [2] in order to facilitate comparison with our results. The rest of variables were for 2016.

Total health spending refers to average expenditures on health per person, expressed in international dollars using purchasing power parity (PPP).

The Human Development Index (HDI) was constructed to measure social development [19]. The index is based on the average of three indicators: life expectancy at birth, years of schooling for adults and expected years of schooling for children, as well as (log) GDP per capita.

One of the operational goals of the United States Agency for International Development (USAID) is to manage crises and to promote stability, recovery and democratic reform. Progress in achieving this goal is monitored also by the change in the ICRG economic and financial risk rating [20]. The United States President’s Malaria Initiative (PMI) is a government program to fund malaria control. PMI is administered by USAID. PMI USAID supports proven, cost-effective prevention and treatment interventions and helps countries scale up access to these interventions nationwide.

The population at risk of malaria transmission was defined as the population living in areas where malaria transmission occurs. The malaria incidence rate was defined as the number of malaria cases per 1000 people in the risk population per year. The rate of malaria incidence proxies the burden of malaria.

The binary variable for malaria transmission was set as 0 (for no transmission) in countries with the zero malaria incidence and as 1 (for present transmission) in countries with the malaria incidence greater than zero. The *Plasmodium vivax* species was calculated as the proportion of *Plasmodium vivax* to all *Plasmodium* species.

The malaria national intervention policy and strategy adoption was transformed to binary variables. The variable was set to 1 if the policy was implemented or to 0 if the policy was absent. The use of intermittent preventive treatment in pregnancy (IPTp) has no recommendation for countries outside of Africa [21], thus values for non-African countries were imputed to 0 (as absent). Missing values were imputed to 0 (as absent).

If per capita data for variable were not available, the corresponding values were calculated using the total population.

The latitude of the country centroid was converted to absolute value. The binary variable indicating a small country was set to 1 for countries fulfilling the small-country criteria, and to 0 for the rest of the countries. The binary variable for being landlocked was set to 1 for countries with no access to the sea, and to 0 for the rest of the countries.

Explanatory variables were chosen based on the available evidence or were derived from plausible hypotheses. Environmental parameters (e.g. latitude and elevation), social and economic parameters (e.g. GDP and urbanization) and institutional parameters (e.g. aid funding) were measured using variables at the country level.

### Data collection

Hypotheses were tested using a dataset with countries as observational units. The data at the country level were collected from the WHO, the World Bank, the Center for International Development and other public sources (Additional file 2). The data quality control of the primary sources was assumed sufficient to avoid missing data or selection bias.

### Data analysis

Continuous and binary variables were compared using two sample t-tests. Statistical significance was determined using a two-sided significance level of α = 0.05. Predictors of malaria outcome were estimated by regression analysis with the multiple linear regression model:

$$ \mathrm{Y}={\beta}_0+{\beta}_1{\mathrm{X}}_1+{\beta}_2{\mathrm{X}}_2+\gamma \left({\mathrm{X}}_1\ast {\mathrm{X}}_2\right)+\epsilon $$

and binary logistic regression model: logit(Y) =  β_0_ + β_1_X_1_ + β_2_X_2_ + γ(X_1_*X_2_) + ε . Interaction term (X_1_*X_2_) and possible confounding factors were included in regression models. The data were checked for multicollinearity. The goodness of model fit was measured by the R^2^ or pseudo R^2^ statistic as appropriate.

Consistency checks and statistical analyses were performed using Stata (release 14; StataCorp).

## Results

### Descriptive statistics

The majority of variables were found to have a skewed distribution and were transformed by either taking the logarithm or square root. Continuous variables are listed in Table 1 and categorical variables are listed in Table 2. The basic variable characteristics are described in terms of means and standard deviations for continuous variables and counts and proportions for categorical variables.

**Table 1:** Summary statistics of continuous variables

Variable^a^	Units	N	Mean	SD	Min	Max
Land area	log of km^2^	215	4.72	1.31	0.30	7.22
Latitude of country centroid	absolute degree	164	27.35	17.78	0.42	74.70
Elevation above sea level	mean m	164	626.92	560.98	9.17	3185.92
Population size	log	215	6.59	1.06	4.05	9.13
Population density	log	195	1.9	0.62	0.29	4.41
Urban population	proportion	215	0.6	0.24	0.12	1.00
Population within 100 km of coast	proportion	164	0.43	0.36	0.00	1.00
GDP per capita PPP	log of US$	192	4.07	0.5	2.9	5.06
Total GDP PPP	log of millions US$	192	10.79	1.04	7.62	13.28
Total health expenditure per capita PPP	log of intl $	185	2.83	0.57	1.47	3.99
Government health to total government expenditure	proportion	186	3.56	2.34	0.37	13.06
Official development assistance received per capita	current US$	139	124.24	318.11	−2.34	3034.15
Openness (Sachs&Warner)	–	139	0.25	0.40	0.00	1.00
International country risk guide index	–	98	5.68	2.26	2.27	9.98
HDI	–	188	0.69	0.15	0.35	0.94
Malaria incidence	log of 1000 population at risk	105	0.92	1.41	−1.30	2.66
Malaria incidence	1000 population at risk	105	96.03	129.49	0.05	460.90
*Plasmodium vivax* species	proportion	105	0.27	0.37	0.00	1.00
Global Fund funding for malaria control per capita	US$	90	0.88	1.82	−0.07	14.85
PMI USAID funding for malaria control per capita	US$	90	0.27	0.55	0.00	3.10
World Bank funding for malaria control per capita	US$	91	0.01	0.03	−0.01	0.20
UK government funding for malaria control per capita	US$	90	0.04	0.20	0.00	1.29
Government funding for malaria control per capita	US$	70	0.80	2.98	0.00	24.89
UNICEF funding for malaria control per capita	US$	61	0.03	0.13	0.00	1.04
Policy score 1 (from factor analysis)	–	93	0.08	0.40	−0.69	0.82
Policy score 2 (from factor analysis)	–	93	0.73	0.39	−0.47	1.44
Policy score 3 (from factor analysis)	–	93	−0.18	0.40	−0.87	0.92
Policy score 4 (from factor analysis)	–	93	1.02	0.39	−0.05	1.84

^a^ *GDP* gross domestic product; *HDI* human development index; *Global Fund* Global Fund to Fight AIDS, Tuberculosis and Malaria; *NMCP* national malaria control programme; *PMI* United States President’s Malaria Initiative; *PPP* purchasing power parity; *UK* United Kingdom of Great Britain and Northern Ireland; *UNICEF* United Nations Children’s Fund; *USAID* United States Agency for International Development; *log* common logarithm. Funding for malaria control as reported by donors (except UNICEF)

**Table 2:** Summary statistics of categorical variables

Variable ^a^	N	Proportion of countries
Small country dummy	215	0.35
Landlocked country dummy	149	0.23
Island dummy	96	0.64
Malaria transmission dummy	215	0.47
ITNs/LLINs are distributed free of charge	93	0.89
ITNs/LLINs are distributed to all age groups	93	0.75
ITNs/LLINs are distributed through mass campaigns to all age groups	93	0.75
IRS is recommended by malaria control programme	93	0.90
DDT is used for IRS	93	0.10
IPTp is used to prevent malaria during pregnancy	93	0.40
Seasonal malaria chemoprevention (SMC or IPTc) is used	93	0.11
Patients of all ages should get diagnostic test	93	0.99
Malaria diagnosis is free of charge in the public sector	93	0.86
RDTs are used at community level	93	0.56
G6PD test is recommended before treatment with primaquine	93	0.19
ACT for treatment of *Plasmodium falciparum*	93	0.99
Pre-referral treatment with quinine or artemether IM or artesunate suppositories	93	0.59
Single dose of primaquine is used as gametocidal medicine for *Plasmodium falciparum*	93	0.47
Primaquine is used for radical treatment of *Plasmodium vivax* cases	93	0.57
Directly observed treatment with primaquine is undertaken	93	0.29

^a^ *ACT* artemisinin-based combination therapy; *DDT* dichloro-diphenyl-trichloroethane; *G6PD* glucose-6-phosphate dehydrogenase; *IM* intramuscular; *IPTc* intermittent preventive treatment in children; *IPTp* intermittent preventive treatment in pregnancy; *IRS* indoor residual spraying; *ITN* insecticide-treated mosquito net; *LLIN* long-lasting insecticidal net; *RDT* rapid diagnostic test; *SMC* seasonal malaria chemoprevention

### Analysis of economic variables

The Pearson correlation coefficient (0.62; *p* < 0.05) suggests a moderately positive and significant correlation between GDP per capita and the absolute latitude of the country centroid. The Pearson correlation coefficient (− 0.26; *p* < 0.05) shows a weak but significant negative correlation between GDP per capita and the elevation of the country. Landlocked countries have a lower GDP per capita [diff = − 0.34 log US$ (− 0.13;-0.54); *p* < 0.001]. The per capita GDP of island countries is significantly higher [diff = 0.30 log US$ (0.07;0.52); *p* < 0.001] than that of continental countries.

Countries with a greater share of the population living within one hundred kilometres of the coast have higher GDP per capita (Pearson correlation coefficient, 0.36; *p* < 0.05). Landlocked countries have significantly lower GDP per capita. The proportion of the urban population is strongly positively correlated (0.72; *p* < 0.05) with GDP per capita. Prominent exceptions (outliers) are Lichtenstein, the Channel Islands and Trinidad and Tobago.

Openness is moderately but significantly positively correlated (0.59; *p* < 0.05) with the level of GDP per capita. The ICRG index shows a similar relation: countries with established public institutions have a significant and strong positive correlation (0.73; *p* < 0.05) with GDP per capita. GDP per capita is significantly and strongly positively related (0.95; *p* < 0.05) to total health expenditure per capita. GDP per capita is weakly but significantly positively correlated (0.27; *p* < 0.05) with the proportion of government health expenditure to total government expenditure. Total health expenditure per capita and the ICRG index are strongly and significantly related (0.75; *p* < 0.001).

### Analysis of malaria burden

Malaria burden is moderately but significantly negatively correlated (− 0.67; *p* < 0.05) with the absolute latitude of the country centroid. Malaria burden seems to diminish in countries beyond (absolute) 40 degrees of latitude, whereas pre-intervention distribution of malaria reached a maximum of 64 degrees north [22]. Malaria burden is weakly but significantly negatively correlated (− 0.23; *p* < 0.05) with country elevation. The malaria burden on islands is not significantly different [diff = − 1.29 (− 5.50;2.91); *p* = 0.53] from that of landlocked countries.

**Fig. 2:**
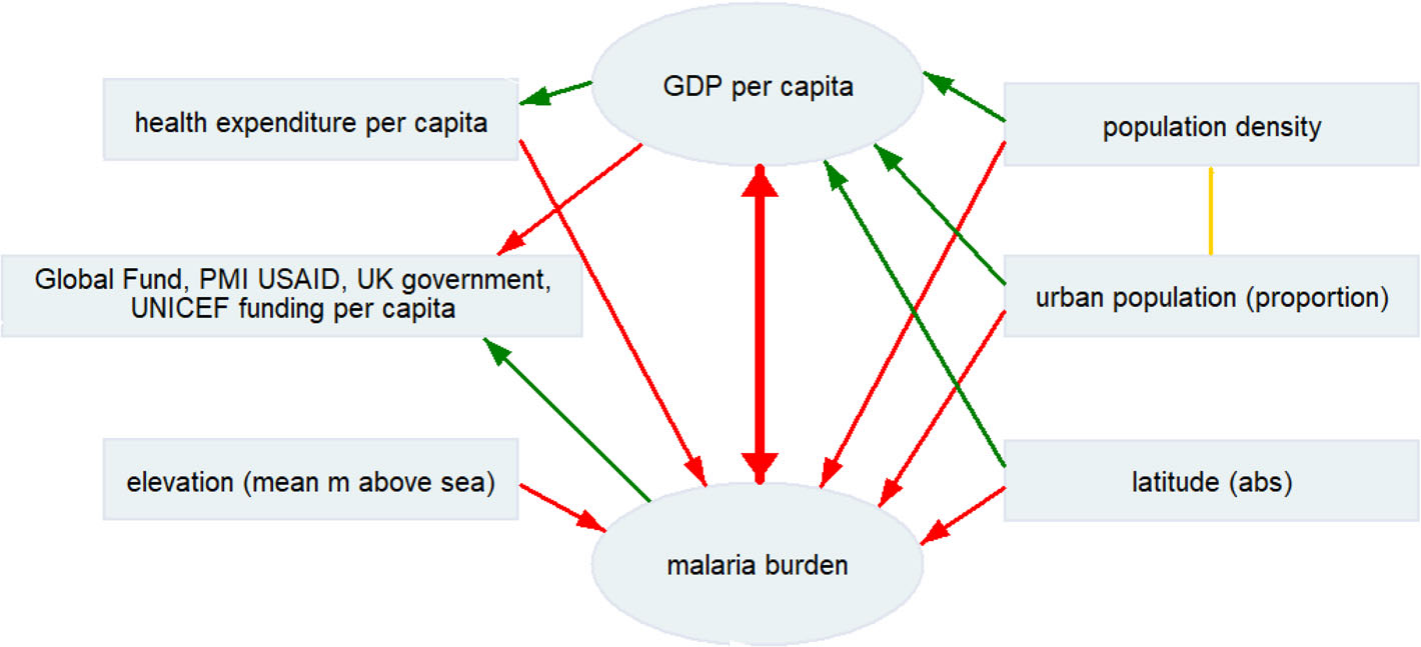
Relationships of malaria burden and social, economic and environmental parameters. * green line indicates a positive correlation, red line indicates a negative correlation, yellow line indicates an interaction between parameters. GDP: gross domestic product; HDI: human development index; PMI: United States President’s Malaria Initiative; UK: United Kingdom of Great Britain and Northern Ireland; UNICEF: United Nations Children’s Fund; USAID: United States Agency for International Development

The burden of malaria is significantly and strongly negatively correlated (− 0.70; *p* < 0.05) with GDP per capita (see Fig. 2). Malaria burden is significantly and strongly negatively correlated (− 0.74; *p* < 0.05) with total expenditure on health per capita. Malaria burden is significantly and moderately negatively correlated (− 0.44; *p* < 0.05) with the proportion of the population living in cities.

Malaria burden is significantly moderately positively correlated (0.49; *p* < 0.05) with PMI USAID funding for malaria control per capita. Global Fund funding for malaria control per capita is significantly but weakly negatively correlated with both land area (− 0.33; *p* < 0.05) and population size (− 0.37; *p* < 0.05). Government NMP funding for malaria control per capita is significantly and moderately negatively correlated (− 0.53; *p* < 0.05) with population size, with Sao Tome and Principe as clear outliers. The relationship with the land area is similarly significant (− 0.46; *p* < 0.05). PMI USAID, World Bank, UK government, and UNICEF funding are not significantly correlated with land area or population size.

### Analysis of malaria predictors 

The probability of malaria transmission increases significantly with country size (*p* < 0.01) and significantly decreases with an increase in the latitude and GDP per capita (*p* < 0.01, Table 3).

**Table 3:** Logistic regression model of any malaria transmission

	any malaria transmission
**(unit)**	**(95% CI)**
Land area	1.531
(log km^2^)	(0.688–2.374)**
Latitude of country centroid	− 0.151
(abs)	(−0.210 - -−0.091)**
GDP per capita	−3.099
(log US$)	(−4.514 - −1.685)**
Constant	7.146
	(1.482–12.811)*
Pseudo R^2^	0.68
*N*	157

* *p <* 0.05; ** *p* < 0.01; *CI*: confidence interval, dependent variable: any malaria transmission; malaria transmission: malaria incidence greater than zero

The malaria burden significantly decreases (*p* < 0.05) with population density and significantly decreases with the latitude (*p* < 0.01), elevation (*p* < 0.05), and GDP per capita (*p* < 0.01). The results remains significant in the model controlled for a small country (Table 4).

**Table 4:** Multiple linear regression model of malaria burden

	malaria burden (log)	malaria burden (log) with small country dummy
**(unit)**	**(95% CI)**	**(95% CI)**
Population density	−0.349	−0.351
(log)	(−0.669 - -0.030)*	(−0.669 - −0.007)*
Latitude of country centroid	−0.067	−0.067
(abs)	(−0.081 - -0.052)**	(−0.080 - -−0.051)**
Elevation	−0.022	0.022
(mean m above sea level, square root)	(−0.040- -0.004)*	(−0.040- -−0.004)*
GDP per capita	−1.498	−1.497
(log US$)	(−1.854 - -1.142)**	(− 1.857 - -−1.137)**
Small country	–	0.013
(dummy)		(−0.622–0.595)
Constant	7.484	7.489
	(6.004–8.595)**	(5.980–8.601)**
R^2^	0.698	0.694
N	91	91

* *p* < 0.05; ** *p* < 0.01; *CI*: confidence interval; dependent variable: malaria burden

### Analysis of country size

Small countries do not differ significantly from large countries in their malaria burden [diff = 0.28 (− 2.86; 3.42); *p* = 0.85]. Being a small country is significantly positively strongly correlated with being an island, is moderately correlated with official development assistance received per capita and Global Fund and government NMP funding for malaria control per capita, and is weakly correlated with population density. Being a small country is significantly and strongly negatively correlated with population size and land area, and is weakly correlated with PMI USAID funding per capita.

### Malaria intervention policy and strategy adoption

Binary indicators for malaria national intervention policies and strategy adoption were used (Table 5) for the factor analysis. From the analysis, factors 1 to 6 which had eigenvalues above one, account for 89% of the total variation. Factor 1 and factor 2 did not show any relevant clustering. Their highest loadings exceed the absolute value of 0.70, which is usually a strong result. Factors 1–4 have at least three variables with loadings above 0.50. The relationships among the factors, malaria burden, and the social, economic and environmental variables using a heat map are visualized in the Additional file 3. The factor values were carried over into new policy scores.

**Table 5:** National intervention policy and and strategy adoption with factor loadings

National intervention policy and strategy adoption	Factor1	Factor2	Factor3	Factor4	Factor5	Factor6
ITNs/LLINs are distributed free of charge	-0.31	-0.22	-0.59	0.56	0.32	0.06
ITNs/LLINs are distributed to all age groups	0.03	0.58	-0.11	0.66	0.01	-0.40
ITNs/LLINs distributed through mass campaigns to all age groups	-0.47	0.41	-0.5	0.18	0.29	-0.31
IRS is recommended by malaria control programme	-0.16	0.68	0.11	-0.47	0.19	-0.34
DDT is used for IRS	-0.03	0.57	0.47	-0.04	0.61	0.25
IPTp is used to prevent malaria during pregnancy	-0.66	0.05	0.6	0.38	-0.15	0.06
Seasonal malaria chemoprevention (SMC or IPTc)	-0.67	-0.08	0.65	0.26	-0.02	-0.02
Patients of all ages should get diagnostic test	0.55	0.01	0.66	0.5	-0.05	0.03
Malaria diagnosis is free of charge in the public sector	0.67	0.21	0.05	0.33	0.43	0.34
RDTs are used at community level	-0.41	0.35	-0.43	-0.11	-0.07	0.61
G6PD test is recommended before treatment with primaquine	0.40	0.25	-0.31	0.49	-0.54	0.11
ACT for treatment of *Plasmodium falciparum*	-0.40	0.82	0.08	-0.08	-0.37	0.02
Pre-referral treatment with quinine or artemether IM or artesunate supp	-0.70	0.32	-0.19	0.15	-0.12	0.21
Single dose of primaquine is used as gametocidal medicine for *Plasmodium falciparum*	0.68	0.53	-0.01	-0.05	0.02	0.10
Primaquine is used for radical treatment of *Plasmodium vivax*	0.91	0.01	-0.17	0.11	0.05	-0.13
Directly observed treatment with primaquine is undertaken	0.66	0.43	0.10	-0.22	-0.29	0.04

*ACT artemisinin-based combination therapy, *DDT* dichloro-diphenyl-trichloroethane, *G6PD* glucose-6-phosphate dehydrogenase, *IM* intramuscular, *IPTc* intermittent preventive treatment in children, *IPTp* intermittent preventive treatment in pregnancy, *IRS* indoor residual spraying, *ITN* insecticide-treated mosquito net, *LLIN* long-lasting insecticidal net, *RDT* rapid diagnostic test, *SMC* seasonal malaria chemoprevention; factor loadings: factor 1-6

### Policy scores

Policy score 1 is moderately positively correlated with the HDI (0.69; *p* < 0.05), GDP per capita (0.66; *p* < 0.05), total health expenditure per capita (0.66; *p* < 0.05), weakly correlated with being an island (0.49; *p* < 0.05), urban population share (0.46; *p* < 0.05), the latitude (0.41; *p* < 0.05), the share of the population within 100 km of the coast (0.38; *p* < 0.05), and the proportion of government health expenditure to total government expenditure (0.36; *p* < 0.05) and economic openness (0.23; *p* < 0.05). Policy score 1 is significantly negatively and strongly correlated with the malaria burden (− 0.78; *p* < 0.05), moderately with PMI USAID funding (− 0.57; *p* < 0.05), weakly with Global Fund aid (− 0.38; *p* < 0.05), World Bank aid (− 0.20; *p* < 0.05), UK government funding (− 0.31; *p* < 0.05), and UNICEF funding (− 0.36; *p* < 0.05) per capita and being a landlocked country (− 0.23; *p* < 0.05). Policy score 1 is not significantly related to government NMP funding per capita or being a small country.

**Fig. 3:**
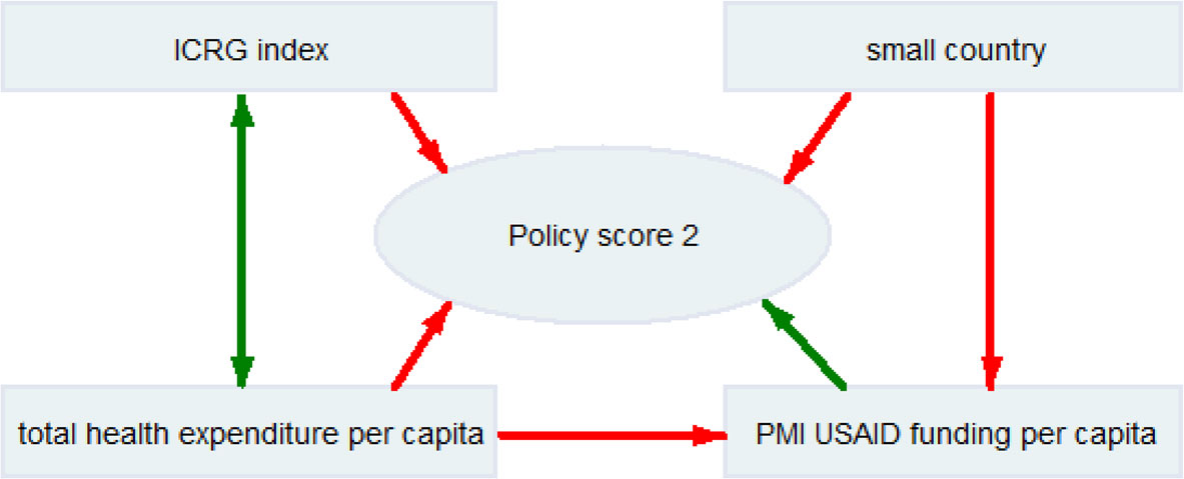
Relationships of policy score 2 and economic, institutional and environmental parameters. * green line indicates a positive correlation, red line indicates a negative correlation with policy score 2. ICRG: the international country risk guide; PMI: United States President’s Malaria Initiative; USAID: United States Agency for International Development

Policy score 2 has weak but significant relationships (see Fig. 3) with the ICRG index (− 0.26; *p* < 0.05), PMI USAID funding per capita (0.21; *p* < 0.05) and being a small country (− 0.27; *p* < 0.05). Policy score 2 is not significantly correlated with GDP per capita or the malaria burden.

Policy score 3 was significantly weakly negatively correlated with the proportion of *Plasmodium vivax* (− 0.27; *p* < 0.05) and elevation (− 0.24; *p* < 0.05). Since the prevalence of *Plasmodium falciparum* outside Africa is generally below 5%, policy score 3 could also be considered as representing malaria policy outside of Africa. Policy score 4 was significantly and weakly correlated (0.26; *p* < 0.05) with openness.

## Discussion

Access to the sea strengthens economic development by lowering transport costs. Coastal regions are the core of a country’s economic growth and its development of agglomerations and urbanization. Many landlocked countries are poor, and many coastal countries are rich, particularly island economies, which have the easiest access to the sea. Being geographically located closer to the sea possibly influences the mean elevation enough to cause a relationship between elevation and economic output.

Countries nearer the equator have a lower GDP per capita. Our analysis shows that smaller countries do not face economic disadvantages: GDP per capita decreases with country size. GDP per capita is higher in urbanized countries and countries with a higher population density. Population density and urbanization seem to be mutually influential and interrelated. Open trade policies are usually found to improve economic development in the long term [23]. Our data support the claim that openness and stable public institutions are associated with a higher GDP per capita. Countries with higher GDP spend a greater part of their GDP on the health system and have higher total health expenditure per capita as well.

The ratio of government health expenditure to total government expenditure increases with GDP. Thus, higher GDP is associated with higher health expenditure per person both relatively and absolutely. Governments of countries with higher GDP per capita spend more on health per person.

The malaria burden is higher in tropical countries and countries with lower elevations. These results reinforce previous findings that climate, particularly temperature and rainfall, affects the ability of malaria parasites and vectors to coexist long enough for transmission to occur [24]. Transmission stops at elevations above 2000 to 2500 m, depending on latitude. There can be other epidemiologically important environmental transmission parameters beyond elevation [25]. The malaria burden of islands is not significantly different from that of continental countries. This evidence does not support the general belief that eliminating malaria on islands is easier than eliminating malaria on continents [26]. Total health expenditure per capita is related to a lower malaria burden. If the total expenditure per person is small, it may negatively influence the allocation of financial resources to malaria control. If the total amount is high enough, malaria control does not interfere with other health priorities [26]. Governmental investment in malaria control does not seem to increase knowledge about malaria [10]. Rural areas exhibit a higher malaria burden. Our findings support previous reports that urbanization affects vectors and reduces their biological ability to carry and transmit the disease [27] Therefore, people in urban areas are less likely to be infected with malaria. However the opposite effect has also been observed at the micro level. Malaria burden is most strongly correlated with HDI. GDP per capita combined with the country’s longevity and education indicators is more strongly related to malaria burden than GDP per capita alone.

Our analysis supports previous findings [28] of the relation between malaria and GDP. The previous discussion underlines the diminishing effects of malaria on economic development, indicating that the effective control of malaria can positively impact economic development. Since results have a significant bidirectional association (see Fig. 2). a stronger economy could strengthen also malaria control. The complex ecology of the vector at the micro level could contribute to this bidirectional causality.

Malaria burden could serve as a proxy for other tropical diseases with similar vector conditions, such as dengue fever [24].

### International funding

To a vast degree, the commitment of international agencies determines the success of malaria control programmes in the long term. International aid funding accounts for approximately 68% of funds globally. The largest share of malaria funding is provided by The Global Fund (40% of total funding), followed by the USAID President’s Malaria Initiative (PMI) (26%), the UK government (7%) and the World Bank (3%). Both the UK government and the PMI USAID also contribute through the Global Fund. Multinational organizations have boosted their support of the global health agenda so that their contributions are higher than those of organizations that were specifically established to promote the global health agenda such as the WHO.

Our results suggest that funding for malaria control per capita to small countries is inconsistently distributed depending on the funding provider. People in smaller countries and in countries with smaller populations benefit disproportionally from malaria control funding. Only PMI USAID funding is related to the institutional quality of the recipient country.

### Policy scores

Policy decisions are being increasingly challenged, and demands for policies to be transparent and data driven are also increasing. Having our results at the country level allows for easy comparisons and could contribute to the optimization of funding resources.

Policy score 1 describes the endemicity of malaria.

Policy score 2 indicates economic and institutional conditions in 93 countries and represents the variation in national malaria intervention policy that is not explained by malaria endemicity (Additional file 4). Since policy score 2 is related to the size of the country without being related to malaria or GDP, this score explains novel components of the small country bias. The quality of institutions and total health expenditure are influenced by corruption. Weakening corruption is fundamental to improving the efficiency of health care systems [29]. Corruption is correlated with public spending on education and health and even with total health expenditure per capita [22]. Improving the quality of national institutions seems to increase averaged personal spending on health [30]. Welfare spending, defined as spending on health, education, and social security, contributes to sustaining peace and is negatively related to the incidence of conflicts [28].

The research on malaria neglects the *Plasmodium vivax* species, and policy score 3 could provide more insight into its endemicity. Policy score 4 represents open trade policies.

### Country size

The high costs of public service, telecommunication and transportation impede the delivery of health care, education and infrastructure services in small countries.. Many governments are implementing health system reforms in order to improve health services and health financing. International agencies support these reforms through various development policies. Environmental health is of growing importance.

Relationships between the malaria burden and the environmental endowments and geographic and social conditions were consistently found at the country level. According to results small countries do not seem to be disadvantaged in the allocation of GDP per capita or face a higher malaria burden than large countries. However, small countries receive more development assistance per capita from international agencies. This difference could be partially explained by the high delivery costs of development assistance due to geographic conditions or by using different allocation criteria.

Many small countries face the re-emergence of vector-borne diseases such as dengue fever. However, according to our results, malaria is more likely to increase in larger countries.

### Limitations

The strength of any data analysis depends on the accuracy of the data. The specific ecosystem of malaria research utilizes data from many sources of various quality. Nevertheless, with the same sources as the WHO uses, the data quality should be reasonable enough to address study questions. Most variables use data sources from the same year, but for some privately owned data sets, the issuing organisations make only older data available or require paid service. Further analyses could extend the group of small countries according to the definition used by the World Bank.

Some explanatory variables could turn out to be proxies for causal parameters such as humidity, rainfall patterns, deforestation or global climatic and ecological changes. Further research could explore trends and causality using time series data and other data that are not publicly available.

## Conclusion

Our study presents the evidence that malaria burden and economic development are bidirectionally related to each other. This finding could be beneficial for the implementation of institutional and health policies: socio-economic development reduces the malaria burden, and at the same time, the control and elimination of malaria supports economic growth. Our findings reveal that international funding for malaria control is inconsistently allocated. The effectiveness of guidelines for malaria control can be improved in the future, and the preference for small countries seems no longer justified.

The small country effect has been firmly established in international developmental policies following claims of small country disadvantages. Our study did not find such conditions to last. The study results suggest, that small countries surpass larger countries in many social and economic resources if parameters are analysed per capita. Health policies are directed specifically to small countries and are determined based on multiple factors. A greater awareness of existing biases can contribute to international efforts to control and eliminate malaria.

Our analysis produced a novel policy index that describes complex relationships between international foreign aid and the socio-economic parameters of recipient countries. This index is independent of malaria endemicity and can provide further insights into health policy development, as well as elucidate the roles of peace building and conflict prevention in global health.

## Supplementary information

**Additional file 1.** Small countries and dependent territories.

**Additional file 2.** Data sources.

**Additional file 3.** Correlation matrix of policy scores, malaria, social, economic and environmental parameters.

**Additional file 4.** Policy score 2.

## Data Availability

The datasets used and/or analysed during the current study are available from the corresponding author on reasonable request.

## Abbreviations

ACT: Artemisinin-based combination therapy
DDT: Dichloro-diphenyl-trichloroethane
G6PD: Glucose-6-phosphate dehydrogenase
GDP: Gross domestic product
Global Fund: Global Fund to Fight AIDS, Tuberculosis and Malaria
HDI: Human development index
ICRG: The international country risk guide
IM: Intramuscular
IPTc: Intermittent preventive treatment in children
IPTp: Intermittent preventive treatment in pregnancy
IRS: Indoor residual spraying
ITN: Insecticide-treated mosquito net
LLIN: Long-lasting insecticidal net
log: common logarithm
NMP: National malaria programme
PPP: Purchasing power parity
PMI: United States President’s Malaria Initiative
RDT: Rapid diagnostic test
SMC: Seasonal malaria chemoprevention
UK: United Kingdom of Great Britain and Northern Ireland
USAID: United States Agency for International Development
UNICEF: United Nations Children’s Fund
WHO: World Health Organization

## References

[1] Dudley L, Montmarquette C (1976). A model of the supply of bilateral foreign aid. Am Econ Rev.

[2] Gallup JL, Sachs JD, Mellinger AD (1999). Geography and economic development. Int Reg Sci Rev.

[3] Easterly W, Levine R (2003). Tropics, germs, and crops: how endowments influence economic development. J Monet Econ.

[4] Braveman PA (2001). Epidemiology and (neo-) colonialism. J Epidemiol Community Health.

[5] Prussing E (2018). Critical epidemiology in action: research for and by indigenous peoples. SSM-Popul Health.

[6] World Health Organization (2016). Action and Investment to Defeat Malaria 2016–2030-for a Malaria-Free World. Technical report, Geneva.

[7] Okorosobo T, Okorosobo F, Mwabu G, Orem JN, Kirigia JM (2011). Economic burden of malaria in six countries of Africa. Eur J Bus Manag.

[8] Williams HA, Durrheim D, Shretta R (2004). The process of changing national malaria treatment policy: lessons from country-level studies. Health Policy Plan.

[9] Online Browsing Platform (OBP) [Internet]. Available from: https://www.iso.org/obp/ui. Accessed 8 May 2020.

[10] Hagenlocher M, Castro MC (2015). Mapping malaria risk and vulnerability in the United Republic of Tanzania: a spatial explicit model. Popul Health Metrics.

[11] Ekström J (2011). The phi-coefficient, the tetrachoric correlation coefficient, and the Pearson-Yule Debate.

[12] Yeomans KA, Golder PA. The Guttman-Kaiser criterion as a predictor of the number of common factors. Statistician. 1982:221–9.

[13] Crowards T (2002). Defining the category of ‘small’states. J Int Dev.

[14] Jalan B. Problems and Policies in Small Countries. Lond Croom Helm. 1982. p. 39–48.

[15] Downes AS. On the statistical measurement of smallness: a principal component measure of country size. Soc Econ Stud. 1988:75–96.

[16] Schiavo-Campo S. Some considerations on development aid to small countries. Dev Policy Small States, Selwyn P (Ed). Croom Helm Lond. 1975.

[17] Sachs JD, Warner A, AAslund A, Fischer S (1995). Economic reform and the process of global integration. Brook Pap Econ Act.

[18] Knack S, Keefer P (1995). Institutions and economic performance: cross-country tests using alternative institutional measures. Econ Polit.

[19] Sagar AD, Najam A (1998). The human development index: a critical review1. Ecol Econ.

[20] Tarnoff C (2015). US Agency for International Development (USAID): Background, operations, and issues. Congressional Research Service Washington, DC.

[21] Global Malaria Programme WHO (2013). Policy Brief for the Implementation of Intermittent Preventive Treatment of Malaria in Pregnancy using Sulfadoxine-Pyrimethamine (IPTp-SP).

[22] Hay SI, Guerra CA, Tatem AJ, Noor AM, Snow RW (2004). The global distribution and population at risk of malaria: past, present, and future. Lancet Infect Dis.

[23] Jain AK. The political economy of corruption. Vol. 2. London: Routledge; 2001.

[24] Dhiman RC, Pahwa S, Dhillon GPS, Dash AP (2010). Climate change and threat of vector-borne diseases in India: are we prepared?. Parasitol Res.

[25] Cox J, Craig M, Le Sueur D, Sharp B (1999). Mapping malaria risk in the highlands of Africa.

[26] Feachem RG, Phillips AA, Hwang J, Cotter C, Wielgosz B, Greenwood BM (2010). Shrinking the malaria map: progress and prospects. Lancet.

[27] Hay SI, Guerra CA, Tatem AJ, Atkinson PM, Snow RW (2005). Urbanization, malaria transmission and disease burden in Africa. Nat Rev Microbiol.

[28] Sachs J, Malaney P (2002). The economic and social burden of malaria. Nature..

[29] Liang L-L, Mirelman AJ (2014). Why do some countries spend more for health? An assessment of sociopolitical determinants and international aid for government health expenditures. Soc Sci Med.

[30] Gupta S, Davoodi H, Tiongson E. Corruption and the provision of health care and education services. In: The political economy of corruption. London: Routledge; 2001. p. 123–53.

