# Supplementary material for: Country size bias in global health: cross-country comparison of malaria policy and foreign aid
Source: Glob Health Res Policy. 2021 Feb 3;6:4. doi: 10.1186/s41256-020-00176-x (PMC7856723; doi:10.1186/s41256-020-00176-x)
Supplement: Supplementary file 1 — Additional file 1. Small countries and dependent territories. [file 41256_2020_176_MOESM1_ESM.pdf]

Additional file 1: Small countries and dependent territories

- American Samoa
- Andorra
- Anguilla
- Antigua and Barbuda
- Aruba
- Bahamas
- Bahrain
- Barbados
- Belize
- Bermuda
- British Virgin Islands
- Brunei
- Cape Verde
- Cayman Islands
- Comoros
- Cook Islands
- Cyprus
- Djibouti
- Dominica
- Equatorial Guinea
- Falkland Islands
- Faroe Islands
- Fiji
- French Polynesia
- Gambia
- Grenada
- Guadeloupe
- Guam
- Guinea-Bissau
- Isle of Man
- Kiribati
- Liechtenstein
- Luxembourg
- Macau
- Maldives
- Malta
- Marshall Islands
- Martinique
- Mauritius
- Micronesia
- Monaco
- Montserrat
- Nauru
- Netherlands Antilles
- New Caledonia
- Niue
- Northern Mariana Islands
- Palau
- Qatar
- Reunion
- Saint Helena
- Saint Kitts and Nevis
- Saint Lucia
- Saint Pierre and Miquelon
- Saint Vincent and the Grenadines
- Samoa
- San Marino
- Sao Tome and Principe
- Seychelles
- Solomon Islands
- Swaziland
- Timor
- Tokelau
- Tonga
- Trinidad and Tobago
- Turks and Caicos Islands
- Tuvalu
- United States Virgin Islands
- Vanuatu
- Wallis et Futuna
