# Supplementary material for: Country size bias in global health: cross-country comparison of malaria policy and foreign aid
Source: Glob Health Res Policy. 2021 Feb 3;6:4. doi: 10.1186/s41256-020-00176-x (PMC7856723; doi:10.1186/s41256-020-00176-x)
Supplement: Supplementary file 2 — Additional file 2. Data sources. [file 41256_2020_176_MOESM2_ESM.pdf]

Additional file 2: Data sources

Variable *	Source	Link
population within 100 kilometres of coast, latitude of country centroid, elevation, landlocked country, openness, international country risk guide index	Center for International Development	www.cid.harvard.edu
land area, population, population density, urban population, total GDP, GDP per capita, government health to total government expenditure, official development assistance received per capita	World Bank	data.worldbank.org/indicator
total health expenditure per capita, malaria incidence per 1000 population at risk, funding for malaria control, ITNs/LLINs are distributed free of charge, ITNs/LLINs are distributed to all age groups, ITNs/LLINs distributed through mass campaigns to all age groups, IRS is recommended by malaria control programme, DDT is used for IRS, IPTp is used to prevent malaria during pregnancy, seasonal malaria chemoprevention (SMC or IPTc) is used, patients of all ages should get diagnostic test, malaria diagnosis is free of charge in the public sector, RDTs are used at community level, G6PD test is recommended before treatment with primaquine, ACT for treatment of *Plasmodium falciparum*, pre-referral treatment with quinine or artemether IM or artesunate suppositories, single dose of primaquine is used as gametocidal medicine for *Plasmodium falciparum*, Primaquine is used for radical treatment of *Plasmodium vivax* cases, directly observed treatment with primaquine is undertaken	World Health Organization	www.who.int /malaria/publications
HDI	United Nations Development Programme	hdr.undp.org/en/data
island	Integrated Island Database	www.island-database.uni-hamburg.de

* ACT: artemisinin-based combination therapy; DDT: dichloro-diphenyl-trichloroethane; G6PD: glucose-6-phosphate dehydrogenase; GDP: gross domestic product; HDI: human development index; IM: intramuscular; IPTc: intermittent preventive treatment in children; IPTp: intermittent preventive treatment in pregnancy; IRS: indoor residual spraying; ITN: insecticide-treated mosquito net; LLIN: long-lasting insecticidal net; NMCP: national malaria control programme; RDT: rapid diagnostic test; SMC: seasonal malaria chemoprevention.
