# Supplementary material for: Country size bias in global health: cross-country comparison of malaria policy and foreign aid
Source: Glob Health Res Policy. 2021 Feb 3;6:4. doi: 10.1186/s41256-020-00176-x (PMC7856723; doi:10.1186/s41256-020-00176-x)
Supplement: Supplementary file 3 — Additional file 3. Correlation matrix of policy scores, malaria, social, economic and environmental parameters. [file 41256_2020_176_MOESM3_ESM.pdf]

Additional file 3: Correlation matrix of policy scores, malaria, social, economic and environmental parameters

	**Variable**	(1)	(2)	(3)	(4)	(5)	(6)	(7)	(8)	(9)	(10)	(11)	(12)	(13)	(14)	(15)	(16)	(17)	(18)	(19)	(20)	(21)	(22)	(23)	(24)	(25)
(1)	Policy score 1	1																								
(2)	Policy score 2	-0.21																								
(3)	Malaria burden	-0.79	0.11																							
(4)	GDP per capita	0.67	-0.15	-0.71																						
(5)	Land area	-0.08	0.15	-0.03	-0.17																					
(6)	Latitude of country centroid	0.41	-0.04	-0.66	0.62	-0.03																				
(7)	Elevation	0.12	0.08	-0.22	-0.26	0.23	-0.02																			
(8)	Population size	-0.02	0.19	-0.06	-0.12	0.85	-0.12	0.11																		
(9)	Population density	-0.06	0.10	0	0.15	-0.64	-0.09	-0.14	-0.18																	
(10)	Urban population	0.47	-0.18	-0.44	0.73	-0.11	0.43	-0.26	-0.13	0.10																
(11)	Population within 100 km of coast	0.39	-0.02	-0.11	0.36	-0.36	-0.09	-0.43	-0.14	0.25	0.40															
(12)	Total health expenditure per capita	0.66	-0.11	-0.75	0.95	-0.14	0.67	-0.23	-0.11	0.11	0.73	0.32														
(13)	Government health expenditure to	0.36	0	-0.17	0.28	-0.12	0.33	-0.02	-0.2	-0.05	0.24	0.13	0.38													
(14)	Official development assistance received	-0.01	-0.05	0.18	-0.01	-0.61	0.11	0.06	-0.72	0.09	0.04	-0.06	0.07	0.21												
(15)	Openness	0.23	-0.05	-0.14	0.59	-0.13	0.27	-0.14	0.07	0.21	0.51	0.36	0.58	0.32	-0.09											
(16)	International country risk index	0.09	-0.26	-0.03	0.73	-0.08	0.71	-0.13	-0.04	0.05	0.53	0.17	0.75	0.35	-0.25	0.69										
(17)	HDI	0.70	-0.12	-0.77	0.95	-0.16	0.68	-0.21	-0.11	0.12	0.69	0.32	0.95	0.35	0.07	0.57	0.71									
(18)	Global Fund funding per capita	-0.39	0.04	0.46	-0.50	-0.33	-0.17	-0.14	-0.37	0.06	-0.27	-0.16	-0.47	-0.04	0.47	-0.23	-0.20	-0.54								
(19)	PMI USAID funding per capita	-0.58	0.21	0.50	-0.49	0.11	-0.24	0.02	0.15	0.09	-0.35	-0.30	-0.45	-0.12	-0.04	-0.17	-0.21	-0.45	0.22							
(20)	World Bank funding per capita	-0.21	-0.17	0.16	-0.15	-0.18	-0.17	-0.06	-0.11	0.12	0.01	0.08	-0.19	-0.05	0.05	-0.08	-0.09	-0.16	0.14	0.14						
(21)	UK government funding per capita	-0.32	0.14	0.25	-0.17	0.01	-0.18	-0.14	0.04	0.16	-0.15	-0.06	-0.24	-0.14	-0.05	0.15	-0.01	-0.19	0.11	0.21	-0.03					
(22)	Government NMCP funding per capita	0.14	-0.08	-0.03	0.11	-0.46	-0.13	-0.08	-0.53	-0.07	0.25	0.09	0.09	0.07	0.52	0.17	0.08	0.06	0.54	-0.06	0.16	0				
(23)	UNICEF funding per capita	-0.36	0.06	0.35	-0.57	0.13	-0.17	-0.01	0.03	-0.08	-0.32	-0.40	-0.50	-0.15	0.01	-0.23	-0.30	-0.40	0.18	0.40	0.15	0.55	0.07			
(24)	Small country	0.13	-0.27	0.10	0.09	-0.73	-0.05	-0.13	-0.84	0.19	0.04	0.15	0.06	0.12	0.57	-0.01	-0.06	0.06	0.25	-0.27	0.06	-0.14	0.48	-0.14		
(25)	Landlocked country	-0.23	-0.08	0.03	-0.29	-0.01	0.06	0.37	-0.18	-0.13	-0.39	-0.68	-0.24	-0.05	0.18	-0.23	-0.07	-0.24	0.23	0.24	0.03	-0.03	-0.02	0.30	-0.1	
(26)	Island country	0.50	-0.13	0.11	0.29	-0.76	-0.32	-0.5	-0.77	0.35	0.31	0.98	0.24	0.16	0.45	0.24	-0.01	0.27	0.19	-0.53	-0.12	-0.16	0.37	-0.43	0.86	-1
