# Supplementary material for: Country size bias in global health: cross-country comparison of malaria policy and foreign aid
Source: Glob Health Res Policy. 2021 Feb 3;6:4. doi: 10.1186/s41256-020-00176-x (PMC7856723; doi:10.1186/s41256-020-00176-x)
Supplement: Supplementary file 4 — Additional file 4. Policy score 2. [file 41256_2020_176_MOESM4_ESM.pdf]

Additional file 4: Policy score 2

Country	Policy score 2
Afghanistan	0.910
Algeria	0.544
Angola	0.317
Argentina	0.544
Bangladesh	1.124
Belize	0.497
Benin	1.234
Bhutan	0.497
Bolivia	0.855
Botswana	0.497
Brazil	0.574
Burkina Faso	1.061
Burundi	-0.108
Cambodia	0.567
Cameroon	0.789
Cape Verde	0.958
Central African Republic	0.057
Chad	0.885
China	0.343
Colombia	1.124
Comoros	1.192
Congo	0.723
Costa Rica	0.813
Cote d'Ivoire	0.231
Democratic Republic of the Congo	0.781
Djibouti	0.058
Dominican Republic	0.524
Ecuador	0.670
El Salvador	1.094
Equatorial Guinea	0.058
Eritrea	1.308
Ethiopia	1.365
French Guiana	0.723
Gabon	0.057
Gambia	0.885
Ghana	0.719
Guatemala	0.986
Guinea	1.058
Guinea-Bissau	0.336
Guyana	0.208
Haiti	0.264
Honduras	0.497
India	1.124
Indonesia	1.124
Iran	1.267
Kenya	0.723

Laos	0.567
Liberia	1.234
Madagascar	0.781
Malawi	0.723
Malaysia	0.764
Mali	0.509
Mauritania	0.490
Mayotte	0.310
Mexico	0.813
Mozambique	1.234
Myanmar	1.124
Namibia	1.267
Nepal	0.779
Nicaragua	0.986
Niger	0.142
Nigeria	0.547
North Korea	0.682
Pakistan	0.284
Panama	0.544
Papua New Guinea	0.820
Paraguay	0.229
Peru	1.439
Philippines	0.937
Rwanda	0.346
Sao Tome and Principe	0.504
Saudi Arabia	0.738
Senegal	1.234
Sierra Leone	1.234
Solomon Islands	0.317
Somalia	1.192
South Africa	0.586
South Korea	-0.472
South Sudan	1.061
Sudan	0.654
Suriname	0.285
Swaziland	0.816
Tanzania	0.454
Thailand	0.772
Timor	1.095
Togo	0.719
Uganda	1.234
Vanuatu	0.779
Venezuela	0.497
Vietnam	0.986
Yemen	0.910
Zambia	1.234
Zimbabwe	1.391
